# Influence of Different Antioxidants on X-Ray Induced DNA Double-Strand Breaks (DSBs) Using γ-H2AX Immunofluorescence Microscopy in a Preliminary Study

**DOI:** 10.1371/journal.pone.0127142

**Published:** 2015-05-21

**Authors:** Michael Brand, Matthias Sommer, Stephan Ellmann, Wolfgang Wuest, Matthias S. May, Achim Eller, Sabine Vogt, Michael M. Lell, Michael A. Kuefner, Michael Uder

**Affiliations:** Department of Radiology, University Hospital Erlangen-Nuremberg, Erlangen, Germany; St. Georges University of London, UNITED KINGDOM

## Abstract

**Background:**

Radiation exposure occurs in X-ray guided interventional procedures or computed tomography (CT) and γ-H2AX-foci are recognized to represent DNA double-strand breaks (DSBs) as a biomarker for radiation induced damage. Antioxidants may reduce the induction of γ-H2AX-foci by binding free radicals. The aim of this study was to establish a dose-effect relationship and a time-effect relationship for the individual antioxidants on DSBs in human blood lymphocytes.

**Materials and Methods:**

Blood samples from volunteers were irradiated with 10 mGy before and after pre-incubation with different antioxidants (zinc, trolox, lipoic acid, ß-carotene, selenium, vitamin E, vitamin C, N-acetyl-L-cysteine (NAC) and Q 10). Thereby, different pre-incubation times, concentrations and combinations of drugs were evaluated. For assessment of DSBs, lymphocytes were stained against the phosphorylated histone variant γ-H2AX.

**Results:**

For zinc, trolox and lipoic acid regardless of concentration or pre-incubation time, no significant decrease of γ-H2AX-foci was found. However, ß-carotene (15%), selenium (14%), vitamin E (12%), vitamin C (25%), NAC (43%) and Q 10 (18%) led to a significant reduction of γ-H2AX-foci at a pre-incubation time of 1 hour. The combination of different antioxidants did not have an additive effect.

**Conclusion:**

Antioxidants administered prior to irradiation demonstrated the potential to reduce γ-H2AX-foci in blood lymphocytes.

## Introduction

The number of imaging and imaging-guided interventional procedures has led to an overall increase of medical radiation exposure[1–3]. Despite elaborate techniques for dose reduction, the advent of more complex interventions in interventional radiology as well as in computed tomography (CT) has increased and therefore radiation exposure remains significant[4–6]. After irradiation DNA damage is caused by oxidative stress induced by free radicals. DNA double-strand breaks (DSB) are the most serious consequence of radiation exposure[7] potentially resulting in chromosomal aberration and carcinogenesis[8,9]. γ-H2AX-foci are recognized to represent DNA double-strand breaks as a biomarker for radiation exposure. To detect these γ-H2AX-foci the H2AX-immunofluorescence microscopy has been established[10–12]. This method is a reliable and sensitive tool for the determination of γ-H2AX-foci induced by computed tomography or angiography[4–7,10,11,13,14]. It is known that a prophylactic administration of antioxidants in mammals, in cell cultures and in human blood lymphocytes leads to a reduction of radiation induced DNA double-strand breaks but to date mainly high radiation exposure was used with a long pre-incubation time or irradiation was administered intravascularly as part of a radioiodine therapy [15–18]. Whether the protective effect of antioxidants was also present at low dose radiation or after a short, clinically relevant pre-incubation time remains unclear. There was only one study that investigated the protective effect of an antioxidant cocktail on radiation-induced DNA DSB in a clinical setting[19]. However this study did not clarify which single antioxidant has the best protective effect, whether all antioxidants have the same pre-incubation time and whether there are synergistic effects at low dose radiation. Also the exact serum concentration of the antioxidant has not been determined[19]. The aim of our study was to establish a dose-effect relationship and a time-effect relationship for individual antioxidants in a randomized, clinically relevant setup. Furthermore we have systematically evaluated the different antioxidants on a potential synergistic effect.

## Materials and Methods

### Setting and participants

The study complies with the Declaration of Helsinki and was performed following local ethic committee approval.

Written informed consent was obtained from every volunteer. Exclusion criteria were: x-ray examination within the last three days, a history of malignant disease (especially lymphoma or leukaemia), radiation therapy or chemotherapy. Blood was obtained from 10 healthy volunteers (mean age: 35.5 years; range 28–50 years, 5 women and 5 men).

For pretesting γ-H2AX-foci were evaluated with EDTA and heparin containing vials in two different concentrations (1.6 mg EDTA/ml blood vs. 3.2 mg EDTA/ml blood; 9 ml S-Monovette; Sarstedt, Nümbrecht, Germany and 16 I.E. Heparin/ml blood vs. 32 I.E. Heparin/ml blood; 4.9 ml S-Monovette Lithium-Heparin 20, Sarstedt, Nümbrecht, Germany) before and after irradiation with 10 mGy.

### Antioxidants

Zinc, trolox, lipoic acid, ß-carotene, selenium, vitamin E, vitamin C, NAC and Q 10 were studied for their effect on the induction of γ-H2AX-foci. For the calculation of the individual concentration we assumed 6,000 mL total blood volume on the basis of 70 ml blood/kg body weight. For the standard concentration we followed the recommended daily intake (RDI) values based on the FDA (Food and Drug Administration), the WHO (World Health Organization) or the package inserts (PI), for further information see Table 1. Based on standard concentration (1 x SC) we tested for each antioxidant a 100 x (1/100 x SC) and a 10 x (1/10 x SC) dilution as well as a 10 x (10 x SC) and a 100 x (100 x SC) concentration.

**Table 1:** Amount of antioxidant substances added to the blood samples adapted to the recommended daily intake (RDI) and a theoretical blood-pool of 6L.

Antioxidants	1 x SC/1 ml blood *	1 x SC/6 l blood†	RDI/PI per day‡
**Zinc** (tablets 20 mg, Verla-Pharm, Germany)	0.0025 mg	15 mg	15 mg
**Trolox** (ampoule 100 mg/ml, Calbiochem, Germany)	0.0167 mg	100 mg	PI (100 mg)
**Lipoic acid** (tablets 600 mg, Meda-Pharma, Germany)	0.1000 mg	600 mg	300–600 mg
**ß-carotene** (hard capsules 5 mg, Gall-Pharma, Austria)	0.001 mg	6mg	6 mg
**Selenium** (ampoule 100 μg/2 ml, Biosyn, Germany)	0.0167 μg	100 μg	30–70 μg
**Vitamin E** (tablets 400 mg (all-rac-alpha-Tocopherolacetal), Ratiopharm, Germany)	0.0666 mg	400 mg	10–15 mg / PI (400 mg)
**Vitamin C** (ampoule 100 mg/5 ml, Rotexmedica, France)	0.0167 mg	100 mg	100–150 mg
**NAC** (ampoule 300 mg/2 ml, Hexal, Germany)	0.0500 mg	300 mg	PI (300 mg)
**Q 10** (hard capsules 100 mg, Allcura Naturheilmittel, Germany)	0.0167 mg	100 mg	30–200 mg

* Concentration of substance used in the experiments per 1 ml blood

**†** Concentration of substance used in the experiments estimated for a blood volume of 6 l

‡ Current RDI (FDA, WHO); PI: recommendation on package insert

### Pre-incubation time, concentration and combination of the antioxidants

Blood samples (27 ml blood/person for each experiment) were taken using EDTA containing vials. All blood samples were incubated and stored at standard conditions (37°C, 5% CO2, 95% air) in 15-mL plastic centrifugation vials (Nunc, Langenselbold, Germany) and stained 5 minutes after irradiation with 10 mGy. All following experiments were repeated 3 times for each of the 10 volunteers.

- Individual baseline levels of γ-H2AX-foci were measured in each sample prior to antioxidant exposure and irradiation.
- Blood samples were pre-incubated with different antioxidants (at 1 x SC) for 4 h, 3 h, 2 h, 1 h, 30 min and 15 min before irradiation to test whether there was a time dependent effect on γ-H2AX-foci induction.
- To evaluate the influence of different concentration levels the blood samples were pre-incubated with different antioxidant concentrations for 1 h.
- To test whether or not the most effective antioxidants had an additive effect we incubated the blood samples of our volunteers for 1 hour at standard conditions with different combinations of two antioxidants (selenium, vitamin E, vitamin C and NAC) at 1 x SC.

### Effect of NAC after pre-incubation in-vitro and in-vivo

To test if there is also an in-vivo effect all volunteers (n = 10) received 300 mg NAC i.v. (repeated 3 times for each volunteer, time interval between repetition 1–2 weeks). Blood samples were drawn immediately before, 15 min, 30 min, 1 h, 2 h, 3 h and 4 h after NAC-injection. Simultaneously blood of these volunteers was incubated in-vitro with NAC (at 1 x SC) for 15 min, 30 min, 1 h, 2 h, 3 h and 4 h. All blood samples were irradiated extracorporeal with 10 mGy and stained after 5 minutes.

### γ-H2AX Immunofluorescence microscopy and irradiation

The method has been described in detail[4,7,10,19,20]. The staining is based on the early phosphorylation of the histone variant H2AX after formation of DSBs. The obtained blood cells were layered onto 6 ml of lymphocyte separation medium 1077 (Biochrom, Berlin, Germany) and centrifuged at 1,200g for 15 min at a temperature of 37°C. The separated lymphocytes were stained overnight using a specific γ-H2AX antibody [dilution 1:2500] (Anti-H2A.X-Phosphorylated (Ser 139), BioLegend, Uithoorn, The Netherlands). After incubation with Alexa Fluor 488-conjugated goat anti-mouse secondary antibody [dilution 1:400] (Invitrogen, Paisley, UK) the blood lymphocytes were mounted with VECTASHIELD mounting medium containing 4,6-diamidino-2-phenylindole (Vector Laboratories, Burlingame, USA).

**Fig 1:**
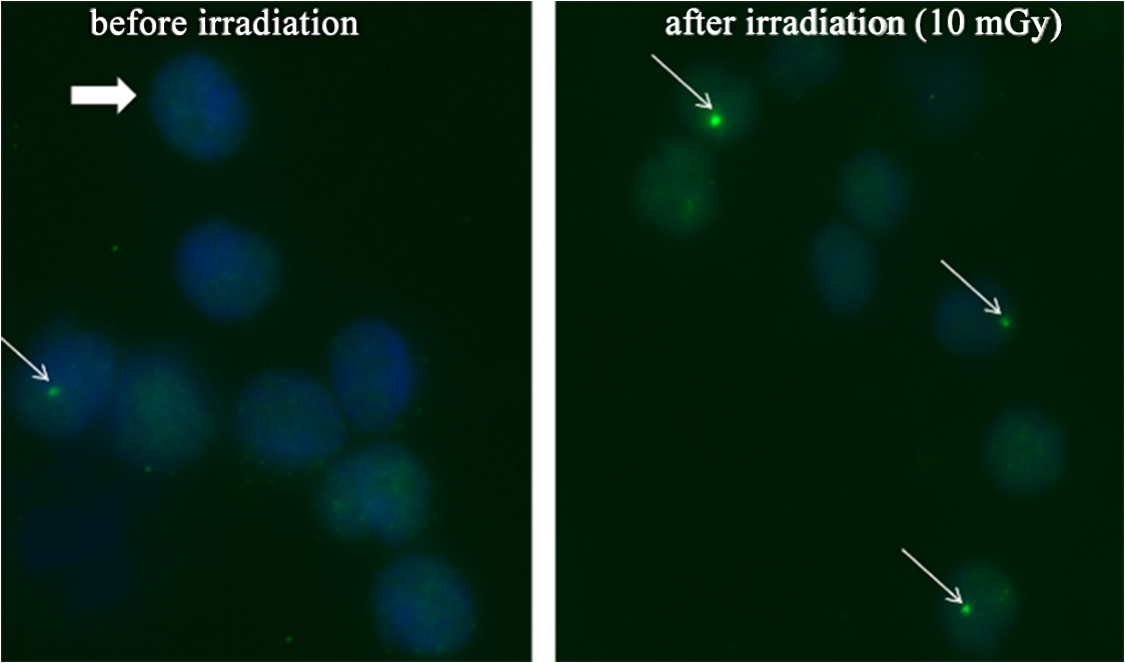
Microscopic image of γ-H2AX foci in human blood lymphocytes before and after irradiation with 10 mGy The tiny green dots represent one γ-H2AX focus (thin arrow) in human blood lymphocytes (thick arrow). On the left image blood lymphocytes before irradiation are depicted, while irradiated (10 mGy) lymphocytes are shown on the right panel.

Fluorescence analyses were performed with a DM 6000 B microscope (Leica, Wetzlar, Germany) equipped with an x63 magnification objective. Regardless the pre-treatment each DNA double-strand break appears as a green dot (so-called absolute γ-H2AX-foci) (Fig 1). Cells were counted until 40 γ-H2AX-foci were detected. Every microscope slide was counted at least three times by two blinded observers independently. With these results we calculated a mean for each blood sample. In order to get the x-ray induced γ-H2AX-foci (so-called excess γ-H2AX-foci) we subtracted the absolute γ-H2AX-foci before irradiation (so-called background foci) from the absolute γ-H2AX-foci after exposure.

Before irradiation dosimetry was performed using a dosimeter (DI4 Dosimentor, PTW, Freiburg, Germany) equipped with ionization chamber (M23331-141, PTW, Freiburg, Germany). Irradiation was performed using an Isovolt Titan x-ray generator (GE Sensing & Inspection Technologies, Ahrensburg, Germany) with 120 kV, 0.4 mA, 2 mm aluminum pre-filtration. For a radiation dose of 10 mGy an irradiation of 12 seconds was necessary. During irradiation cells were cultivated at standard conditions (37°C, 5% CO2, 95% air). The distance between the anode and the object was 50 cm.

### Statistical analysis

Unpaired t-test was performed to compare the foci between the blood samples obtained with EDTA containing vials and heparin containing vials. Dunnett’s test was used to compare the excess foci pre-treated with antioxidants to the excess foci without antioxidants. A p-value <0.05 was considered statistically significant. Statistical analysis was performed using the software Prism 4.03, 2005 (Graph-Pad Software, San Diego, CA).

## Results

**Fig 2:**
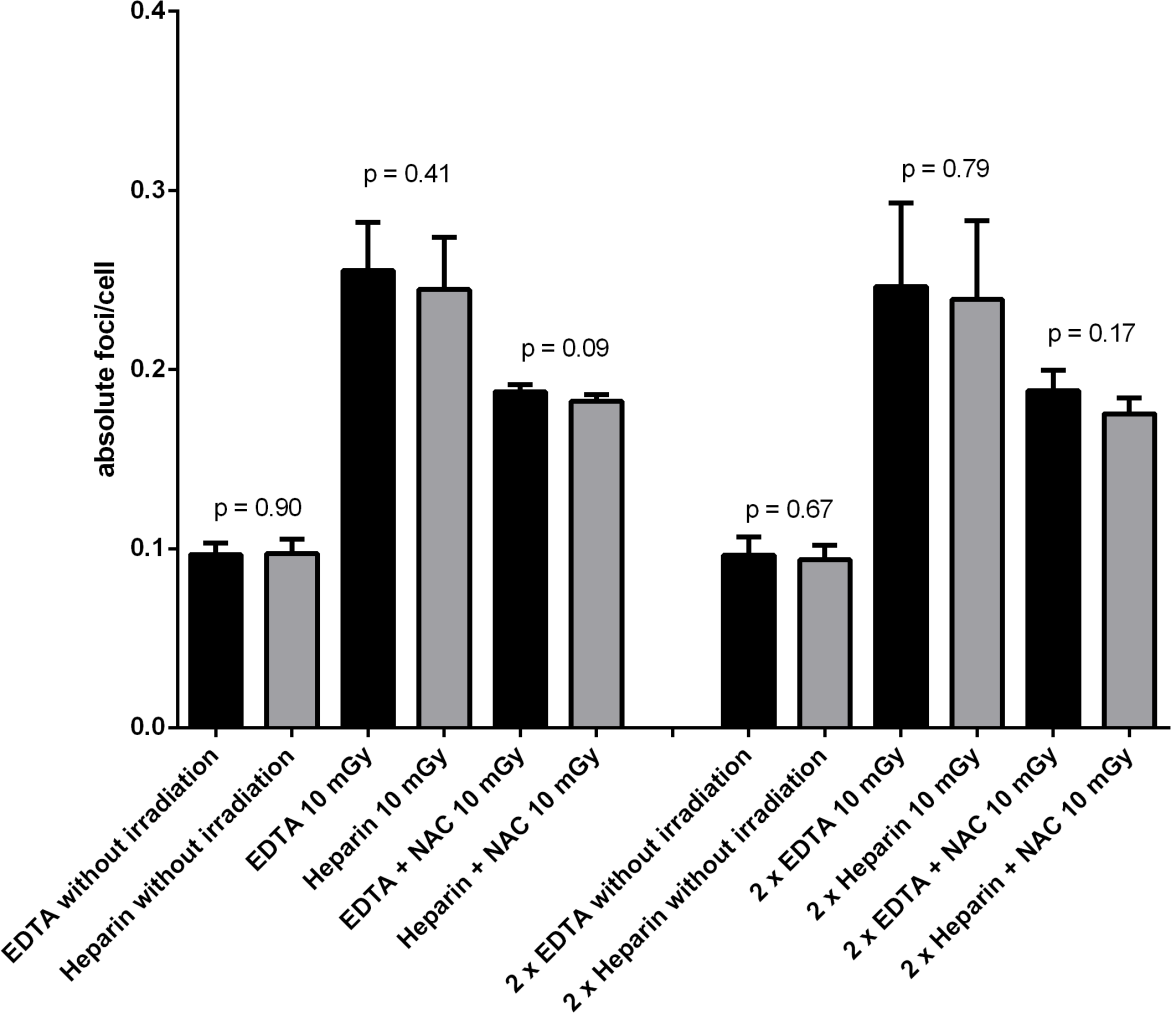
Influence of different vials on DSBs. Influence of EDTA and heparin on γ-H2AX-foci baseline level and γ-H2AX-foci induction after irradiation with 10 mGy. Additionally the effect of 1 hour pre-incubation with NAC (1 x SC) to γ-H2AX-foci/cell (absolute foci) is shown. All values represent mean γ-H2AX-foci/cell (absolute foci) with standard deviations. P-values are shown; the unpaired t-test was used.

The baseline-level of γ-H2AX-foci (so-called background foci) in native (no antioxidant, no irradiation) samples (n = 10) ranged in EDTA vials between 0.083–0.125 γ-H2AX-foci/cell (mean ± standard deviation: 0.096 γ-H2AX-foci/cell ± 0.007) and in heparin vials between 0.085–0.110 γ-H2AX-foci/cell (mean ± standard deviation: 0.097 γ-H2AX-foci/cell ± 0.008) with no significant difference (p = 0.90). Also a double dose of EDTA or heparin showed no significant effect on γ-H2AX-foci/cell (Fig 2; p = 0.67). After irradiation (10 mGy) with and without NAC in 1 x SC (pre-incubation time 1 hour) there was no significant difference between the EDTA containing vials and heparin containing vials (Fig 2). In antioxidant exposed, non-irradiated samples γ-H2AX-foci ranged from 0.082 to 0.123 (mean ± standard deviation: 0.097 DSB/cell ± 0.008). There was no significant difference between the different antioxidants and the different incubation time. Furthermore, no significant difference between native and antioxidant exposed controls (p = 0.2360).

### Pre-incubation time of antioxidants

**Fig 3:**
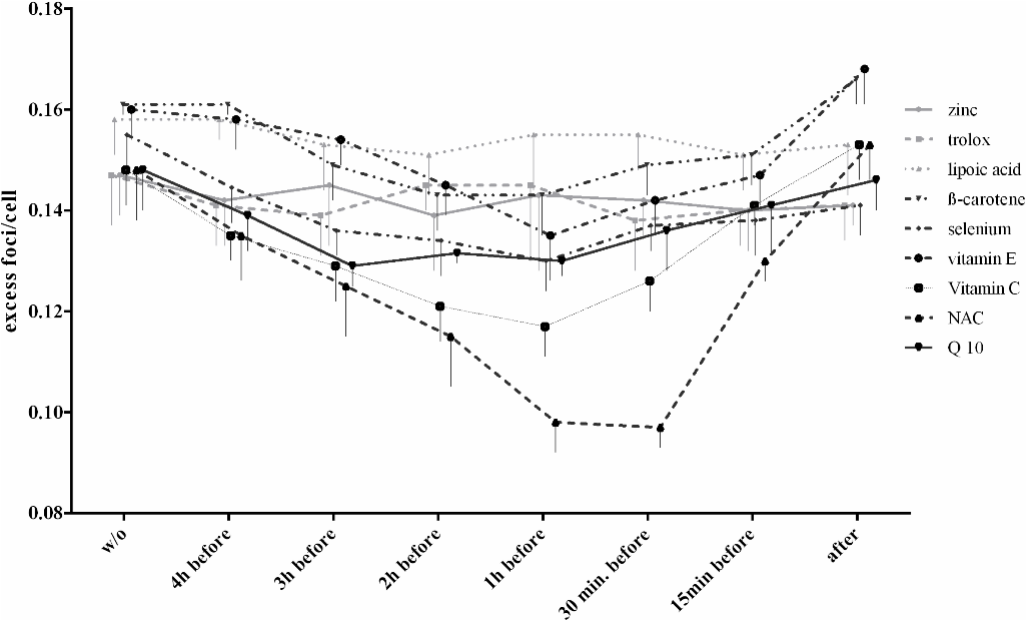
Influence of different pre-incubation times. Dependency of pre-incubation time of the antioxidants (at 1 x SC) on excess foci formation after irradiation with 10mGy. Data represent excess foci/cell with mean ± standard deviation.

Zinc, trolox and lipoic acid (1 x SC) showed no effect on the excess foci at any pre-incubation time. ß-carotene, selenium, vitamin E, vitamin C, NAC and Q 10 showed an incipient reduction of excess foci after 15 minutes with a maximum at 1 hour. With a longer pre-incubation time of 2 to 4 hours there were higher excess foci-levels compared to a 1 h pre-incubation time (Table 2 and Fig 3), but the excess foci-levels are still lower than without antioxidants.

**Table 2:** Time response data for the different antioxidants at 1 x SC (n = 10, repeated 3 times for each volunteer).

Antioxidant	w/o anti-oxidant	4h before	3h before	2h before	1h before	30 min before	15min before	After radiation
**Zinc**	0.147± 0.008	0.142± 0.009	0.146± 0.012	0.138± 0.011	0.143± 0.015	0.143± 0.007	0.139± 0.008	0.141± 0.004
**Trolox**	0.147± 0.010	0.140± 0.008	0.138± 0.007	0.145± 0.005	0.145± 0.015	0.138± 0.010	0.140± 0.007	0.140± 0.007
**Lipoic acid**	0.157± 0.007	0.158± 0.004	0.153± 0.009	0.151± 0.008	0.155± 0.011	0.154± 0.008	0.151± 0.014	0.154± 0.010
**ß-carotene**	0.161± 0.002	0.160± 0.002	0.149± 0.007 *	0.143± 0.007 *	0.143± 0.008 *	0.149± 0.006 *	0.151± 0.006	0.167± 0.005
**Selenium**	0.155± 0.008	0.143± 0.007	0.135± 0.009 *	0.134± 0.007 *	0.130± 0.006 *	0.137± 0.005 *	0.137± 0.007 *	0.139± 0.008
**Vitamin E**	0.159± 0.006	0.155± 0.006	0.154± 0.006	0.144± 0.006 *	0.135± 0.009 *	0.143± 0.006 *	0.147± 0.006	0.168 ± 0.006
**Vitamin C**	0.147± 0.006	0.134± 0.005	0.128± 0.007 *	0.120± 0.007 *	0.117± 0.006 *	0.126± 0.006 *	0.140± 0.004	0.153± 0.007
**NAC**	0.147± 0.010	0.135± 0.010	0.125± 0.010 *	0.115± 0.010 *	0.097± 0.006 *	0.096± 0.004 *	0.130± 0.004 *	0.155± 0.012
**Q 10**	0.148± 0.008	0.139± 0.007	0.128± 0.004 *	0.132± 0.002 *	0.131± 0.003 *	0.136± 0.008	0.141± 0.010	0.147± 0.006

The numbers indicate mean values and standard deviations of x-ray induced γ-H2AX-foci (excess foci)/cell after 10 mGy irradiation.

* indicates a significant deviation from the base level without antioxidant (p <0.05) using Dunnett's test.

### Concentration of antioxidants

**Fig 4:**
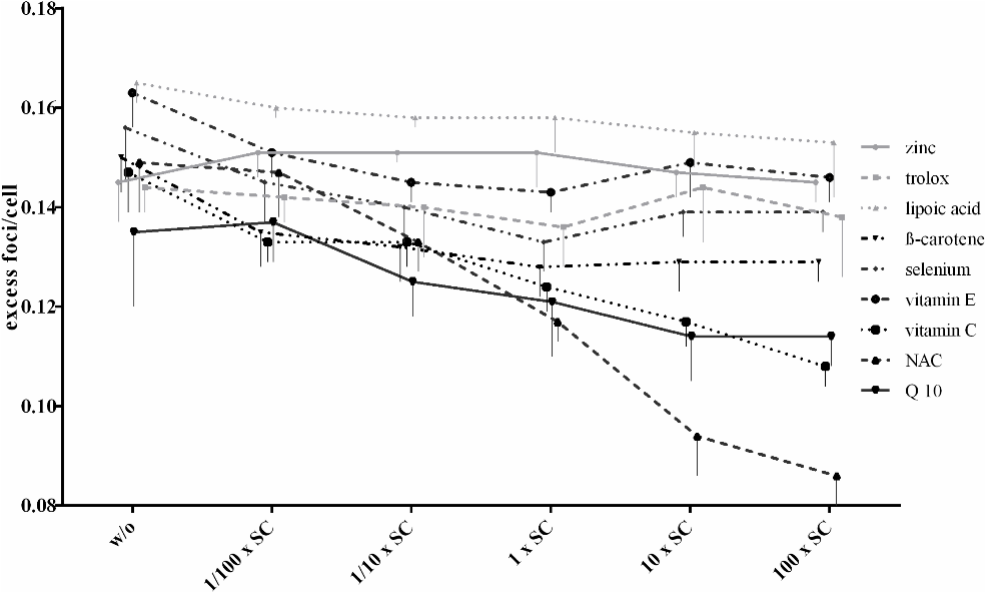
Influence of different concentrations. Dependency of different antioxidant concentrations (1 hour pre-incubation time) on excess foci formation after irradiation with 10mGy. Data represent excess foci/cell with mean ± standard deviation.

Pre-incubation for 1 hour with zinc, trolox and lipoic acid showed no significant decrease of excess foci at any antioxidant concentration (Table 3 and Fig 4) but ß-carotene (15% excess foci-reduction; p < 0.0001), selenium (14% excess foci-reduction; p < 0.0001) and vitamin E (12% excess foci-reduction; p < 0.0001) showed a significant, dose-dependent excess foci-reduction (Table 3 and Fig 4) with a saturation effect at 1 x SC. Q 10 (18% excess foci-reduction; p = 0.0027) showed also dose-dependent significant excess foci-reduction; the maximum effect was achieved at 10 x SC; the 100 x SC showed no further reduction (Table 3 and Fig 4). Pre-incubation with vitamin C (25% excess foci-reduction, p < 0.0001) and NAC (43% excess foci-reduction; p < 0.0001) led to a dose-dependent effect on excess foci reduction with the best effect at 100 x SC.

**Table 3:** Concentration response data for the different antioxidants with 1 hour pre-incubation time (n = 10, repeated 3 times for each volunteer).

Antioxidant	Without antioxidant	1/100 x SC	1/10 x SC	1 x SC	10 x SC	100 x SC
**Zinc**	0.144 ± 0.008	0.150 ± 0.005	0.151 ± 0.004	0.151 ± 0.007	0.145 ± 0.005	0.144 ± 0.004
**Trolox**	0.143 ± 0.005	0.141 ± 0.005	0.139 ± 0.010	0.137 ± 0.008	0.145 ± 0.011	0.137 ± 0.011
**Lipoic acid**	0.164 ± 0.004	0.160 ± 0.002	0.156 ± 0.002	0.157 ± 0.007	0.153 ± 0.007	0.151 ± 0.011
**ß-carotene**	0.150 ± 0.007	0.135 ± 0.007*	0.131 ± 0.007*	0.128 ± 0.006*	0.129 ± 0.006*	0.129 ± 0.004*
**Selenium**	0.155 ± 0.011	0.144 ± 0.008*	0.140 ± 0.007*	0.133 ± 0.006*	0.140 ± 0.005*	0.140 ± 0.004*
**Vitamin E**	0.163 ± 0.007	0.150 ± 0.005*	0.145 ± 0.004*	0.144 ± 0.004*	0.150 ± 0.007*	0.147 ± 0.005*
**Vitamin C**	0.146 ± 0.008	0.133 ± 0.004*	0.133 ± 0.005*	0.125 ± 0.004*	0.118 ± 0.005*	0.109 ± 0.004*
**NAC**	0.150 ± 0.009	0.146 ± 0.009	0.133 ± 0.005*	0.115 ± 0.004*	0.094 ± 0.008*	0.086 ± 0.006*
**Q 10**	0.136 ± 0.015	0.136 ± 0.008	0.124 ± 0.007	0.121 ± 0.011	0.112 ± 0.009*	0.114 ± 0.060*

The numbers indicate mean values and standard deviations of x-ray induced γ-H2AX-foci (excess foci)/cell after irradiation with 10 mGy.

* indicates a significant deviation from the base level without antioxidant (p <0.05) using Dunnett's test.

### Combination of NAC, vitamin C, vitamin E and selenium

**Fig 5:**
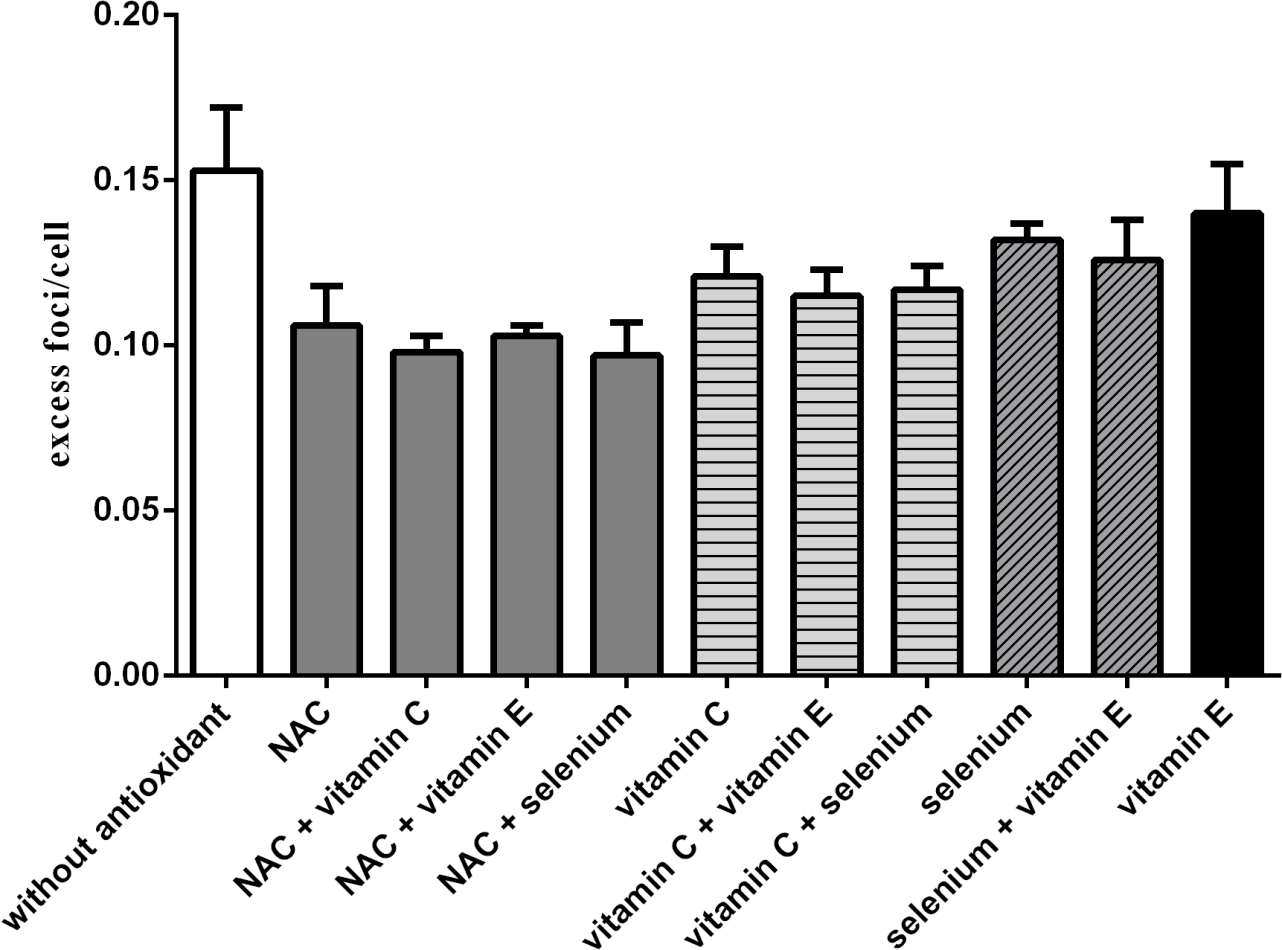
Influence of different antioxidant combinations. Formation of excess foci/cell (mean ± standard deviation) after irradiation with 10mGy for different antioxidant combinations at a pre-incubation time of 1 hour and after irradiation with 10 mGy. No combination resulted in a significant excess foci reduction in comparison to the effect of the best single antioxidant.

The combination of different antioxidants (pre-incubation time 1 hour) did not lead to further significant reduction of excess foci using the standard concentration (1 x SC). Thus, the effect of NAC (0.106 excess foci/cell) could not be increased by a simultaneous addition of vitamin C (0.098 excess foci/cell, p = 0.0813), vitamin E (0.103 excess foci/cell, p = 0.1548) or selenium (0.097 excess foci/cell, p = 0.0691). Additionally the effect of vitamin C (0.121 excess foci/cell) could not be increased by an addition of vitamin E (0.115 excess foci /cell, p = 0.1542) or selenium (0.117 excess foci/cell, p = 0.4808). The combination of selenium (0.132 excess foci/cell) and vitamin E (0.140 excess foci/cell) showed no additive effect (0.131 excess foci/cell, p = 0.1679) (Fig 5).

### NAC pre-incubation in-vitro vs. in-vivo

Identifying NAC as the most promising antioxidant in-vitro, we were able to verify the excess foci reduction when the pre-incubation (injection of 300 mg NAC) occurred in-vivo (0.062 excess foci/cell, 1 h pre-incubation time, p = < 0.05). In direct comparison with the in-vitro incubation we found lower excess foci-level (p = 0.0019) after irradiation with 10 mGy (Table 4) when the pre-incubation took place in-vivo. The excess-foci reducing effect was best when NAC was given 1h prior to irradiation in both experimental settings.

**Table 4:** Comparison of in-vitro and in-vivo application of NAC (1 x SC = 15mg NAC/ml blood) at different time-points prior to and post irradiation (n = 10, repeated 3 times for each volunteer).

	w/o anti-oxidant	4h before	3h before	2h before	1h before	30 min before	15min before	After radiation
in-vitro	0.147 ± 0.020	0.130 ± 0.008	0.118 ± 0.017*	0.115 ± 0.014*	0.097 ± 0.017*	0.096 ± 0.016*	0.130 ± 0.007*	0.155 ± 0.017
in-vivo	0.152 ± 0.010	0.075 ± 0.018*	0.065 ± 0.017*	0.070 ± 0.021*	0.062 ± 0.014*	0.073 ± 0.010*	0.103 ± 0.018*	0.150 ± 0.009

The numbers indicate mean values and standard deviations of excess foci/cell after 10 mGy irradiation.

* indicates a significant deviation from the initial level (p <0.05) using Dunnett's test.

## Discussion

When evaluating DSBs after radiation exposure in human blood lymphocytes, ß-carotene, selenium, vitamin E, vitamin C, NAC and Q 10 reduced the amount of x-ray–induced γ-H2AX-foci whereas zinc, trolox and lipoic acid showed no effect. After low dose radiation NAC had the best protective effect followed by vitamin C. Application of antioxidants 1 hour before irradiation proved to be the most effective incubation time in a clinically relevant setting. The combination of different antioxidant agents did not further reduce the number of excess foci after low radiation exposure. Identifying NAC as the most effective in-vitro antioxidant we also observed a γ-H2AX-foci reducing effect when the pre-incubation took place in-vivo. After irradiation (10 mGy) without pre-incubation with antioxidants an average of 0.14 excess foci/cell was obtained, which is similar to recent studies[7,10,11,14,19].

DSBs are regarded as the most significant DNA lesions induced by ionizing radiation. The immunofluorescence microscopy, based on the early phosphorylation of the histone variant H2AX[7,11], can be used for visualization of foci within cell nuclei representing DNA DSBs[20]. Previous studies have shown that the amount of x-ray–induced DSBs correlate significantly with the radiation dose even after low-level radiation[7,11,13]. 5 minutes after irradiation there is a DSB peak with a following decrease indicating DSB repair[6,7,19]. Therefore, it provides a reliable and sensitive technique for the quantification of x-ray induced DNA damage[6,7,11,12,14,20].

Some previous studies dealt with the different antioxidants in-vitro or in animal studies. Wan XS et al. showed a protective effect of different antioxidants (NAC, Vitamin C, Vitamin E, selenium, Q10 and lipoic acid) on X-ray-induced DSBs in human breast epithelial cells up to a DSB reduction to 87% by single substances and up to 94% using a combination of different antioxidants (Vitamin C, NAC, selenium, Q10 and Vitamin E) [16]. In our study, however, we obtained only a DSB reduction up to 43% for NAC but in contrast we used human blood lymphocytes and a radiation dose which corresponds to standard procedures performed in diagnostic and interventional radiology (10 mGy vs. 50 mGy—2000 mGy). We also obtained a protective effect after pre-incubation with NAC, Vitamin C and Vitamin E, but in contrast we could not find a foci reduction using selenium or lipoic acid and were not able to find a significant additive effect of different antioxidant combinations (NAC, Vitamin C, Vitamin E and selenium). Similar to our results Reliene et al. found a dose-dependent effect of NAC on the induction of H2AX-foci [15] but in contrast we achieved a lower DSB reduction effect (43% vs. 80%). A possible reason might be that Reliene and colleagues have administered NAC to mammals over 10 days so there might be an antioxidant accumulation and the final concentration within the animals was not exactly known. Furthermore, we used low-dose radiation (10 mGy) and not high-dose radiation (1–4 Gy; Reliene et al.) which is a more clinically relevant setting for diagnostic and interventional radiology. Comparing our excess foci-levels for NAC with the excess foci of an antioxidant cocktail from a previous publication[19] the γ-H2AX-foci reduction after 60 minutes is equal (34% vs. 38%). The time-dependent effect of the antioxidants was nearly the same; the best γ-H2AX-foci-redution has been seen after pre-incubation for 60 minutes. There was only a slight difference to the study of Kuefner et al. in early time points (γ-H2AX-foci-reduction of 12% versus 23% after 15 min), probably resulting from an intercellular accumulation of antioxidants due to specific transport mechanisms for the different antioxidants into the cell nucleus following 15 minutes pre-incubation [19].

The different potency of the individual antioxidants cannot be explained completely. Besides selenium all other tested antioxidants act as electron donors which may have a different potency. Thereby vitamin C and vitamin E can be used several times because vitamin C can be recovered by reducing 2 molecules glutathione[21] and similarly, vitamin E can be recovered by reducing vitamin C[22]. Additionally NAC activates and catalyses glutathione-S-transferase which transforms two molecules of glutathione in a dimer to bind free radicals[23,24]. NAC supplies L-cysteine [25] for the cellular synthesis of glutathione and with the help of L-cysteine NAC also activates the enzyme glutathione-transferase-omega[26,27] which is needed to regain reduced vitamin C. Selenium acts as a coenzyme in the active site and increases the reactivity of glutathione-peroxidase[28], which is used to bind free radicals by reduction of two glutathione molecules [29–31]. Despite zinc, trolox and lipoic acid are considered as modifiers of free radicals in biological systems we could not find any significant effects in reducing γ-H2AX-foci.

### Time-dependent effects on γ-H2AX-foci reduction

The best decrease of γ-H2AX-foci was obtained after pre-incubation for 30 to 60 min. This result is in line with previous studies; 1 hour pre-incubation was proved to be most effective using a cocktail of antioxidants[19]. We can only speculate on this observed effect, maybe the antioxidants need some time to enter the cell nucleus to intercept free radicals formed near the DNA. Also a longer pre-incubation time may lead to antioxidant elimination or metabolization. No DSB reduction was observed after adding antioxidants following irradiation. These effects have occurred consistently among individuals and provide evidence that the observed effect of antioxidants on radiation-induced DNA damages is caused by a reduction of γ-H2AX-foci induction and not by accelerated γ-H2AX-foci repair.

### Do “cocktails” improve γ-H2AX-foci reduction?

We did not detect significant additive effects in combining the different substances, although the combination of an electron donor with selenium as an activator of glutathione-peroxidase would suggest an increased or prolonged effect because of different mechanisms inaction. The lack of an additive effect may suggest that all substances affect the same mechanism or at least the same final pathway either directly by trapping free radicals or indirectly by activating enzymes for producing antioxidants to trap free radicals. According to our experiments it is not beneficial to use a cocktail of antioxidants after low level radiation.

### NAC in-vivo

For NAC we were also able to demonstrate a γ-H2AX-foci reduction when the pre-incubation took place in-vivo. With a reduction of 58% of excess foci the results are in line with previous experiments using an antioxidants cocktail in the same setting [19]. However, the extent of γ-H2AX-foci-reduction of NAC was similar when the pre-incubation occurred in-vitro or in-vivo, suggesting that the tested concentrations were in a similar range.

### Limitations

First, the number of individuals studied was small; however, the observed effects were sufficiently large that they achieved statistical significance.

Second, the bio-distribution of orally or intravenously administered antioxidants including first-pass effects, metabolism and elimination cannot be sufficiently modeled and therefore the achieved serum and tissue concentration of the tested substances was not known. The estimation of the blood volume is based on 70 mL blood/kilogram body weight and the average weight of our subjects. We are aware that this is a very rough assessment of the real individual blood volume. What we referred to as standard concentration (SC) is not based on physiologic or toxicological equivalency, but we were following the current recommendations of the FDA, WHO or—if such recommendations do not exist—the proposals of the package insert (PI). The concentration of vitamin E was significantly higher (100 x) than the recommended daily dose, but in this case we have referred to the manufacturer's instructions. Hence some antioxidants could be marginally over- or underdosed but only with minimal effects on the results.

Third, blood samples were irradiated only with 10 mGy, but this seems to be a realistic average radiation dose for cardiovascular interventions, angiography or CT. Previous studies have shown a linear relationship between the radiation dose and the amount of γ-H2AX-foci also for irradiation of the complete blood content [6,7,10,13,19].

Fourth, we only evaluated peripheral blood lymphocytes. Peripheral lymphocytes are differentiated cells without the possibility of cancer induction but these cells could be obtained very easily without major risk for the volunteers. However it has been previously shown in a mouse model that induction of DSBs is comparable between different cell types[12]. But one must consider that, despite the clear reduction of excess foci by some antioxidants, the effect on prevention of cancer still remains unclear.

Fifth, in-vitro experiments can simulate in-vivo processes only to a certain level; hence data is not directly transferable. Therefore clinical studies based on our in-vitro results are the next step.

In conclusion, we were able to establish a dose-effect relationship and a time-effect relationship for individual antioxidants and to demonstrate that different antioxidants have a variable potency to reduce DNA damage after low dose radiation. The best effect was achieved with NAC, although a combination of NAC with other antioxidants led to no further protective effect. Furthermore, we were able to show that for any antioxidant a pre-incubation time of 60 minutes is most effective. Our in-vitro experiments are the basis for further clinical testing and patient studies, but have no direct evidence for cancer induction.
